# The road toward breast cancer single‐disease quality control in China

**DOI:** 10.1002/cai2.93

**Published:** 2023-09-01

**Authors:** Bo Lan, Qiao Li, Fei Ma, Binghe Xu

**Affiliations:** ^1^ Department of Medical Oncology, National Cancer Center/National Clinical Research Center for Cancer/Cancer Hospital Chinese Academy of Medical Sciences and Peking Union Medical College Beijing China

**Keywords:** breast cancer, China, quality control

**Abbreviations**
NCC: National Cancer Center
NHC: National Health Commision
TNM: tumor, node, and metastasis

Malignant tumors have become a major threat to human health worldwide. According to incomplete statistics from the World Health Organization, in 112 of 183 countries, malignant tumors are the primary cause of death among people under the age of 70, and cancer morbidity and mortality are increasing year by year [1]. Owing to the limits of current medical technology, it is impossible to completely overcome this persistent disease.

In China, with the development of the social economy and the advancement of medicine, the diagnosis and treatment of malignant tumors has improved year by year, along with the cure rate of early cancer patients, and the survival period of advanced cancer patients. Statistics from the National Cancer Center of China (NCC) show that the 5‐year survival rate of malignant tumors in China has increased from 30.9% in 2003–2005 to 40.5% in 2012–2015 [2]. China clearly stated in the 2016 *Outline of the Healthy China 2030 Plan* that the health management of chronic diseases for the entire population and the whole life cycle will be realized by 2030, and the overall 5‐year survival rate of cancer will increase by 15%. The 2019 *State Council's Opinions on Implementing the Healthy China Action* clarified this goal and proposed that by 2022 and 2030, the overall 5‐year cancer survival rate should not be lower than 43.3% and 46.6%, respectively. In general, however, problems persist in China, such as uneven diagnosis and treatment levels, inadequate implementation of guidelines, and insufficient sharing of diagnosis and treatment information in the process of cancer diagnosis and treatment. Continuously improving the quality of cancer diagnosis and treatment and standardizing tumor diagnosis and treatment behavior are crucial to improving the cancer survival rate in China.

From the perspective of tumor diagnosis and treatment management, quality control is an important means to achieve the 5‐year survival rate goal. Medical quality control refers to the process of establishing procedures and methods to examine and standardize the reliability and quality of all factors involved in medical work. Medical quality control is important for the guarantee of medical treatment. In recent years, the National Health Commission of the People's Republic of China (NHC) has insisted on carrying out single‐disease treatment quality control in all medical institutions. The NHC issued the *Notice on Further Strengthening the Quality Management and Control of Single Diseases* in 2020 and the *2021 National Medical Quality and Safety Improvement Targets* in February 2021, both of which included quality control indexes for standardized diagnosis and treatment of malignant tumors.

Breast cancer is currently the most common malignant tumor in the world. In 2020, there were 2.26 million new cases of breast cancer worldwide accounting for approximately 11.7% of new cancer cases [1]. In China, breast cancer ranked first among female malignant tumors in China. There were 416,000 new breast cancer cases and 117,000 deaths in 2020 [3]. Breast cancer now has become a prominent disease endangering women's health in China. Although the level of breast cancer diagnosis and treatment in China is constantly improving, there are still problems, such as uneven diagnosis and treatment levels, poor homogeneity, and so on in the diagnosis and treatment of breast cancer in medical institutions in different regions. Therefore, improving the overall survival rate of breast cancer patients in China is inseparable from strengthening the standardized diagnosis and treatment of breast cancer in medical institutions at all levels, and standardized diagnosis and treatment depends on the development of medical quality control management.

## 1 CONSTRUCTION OF A BREAST CANCER QUALITY CONTROL SYSTEM

To further strengthen the national breast cancer single‐disease diagnosis and treatment quality control management, on August 3, 2018, the NCC and National Cancer Quality Control Center established the first single‐disease quality control expert committee: the National Cancer Quality Control Center Breast Cancer Expert Committee. Its members include more than 50 well‐known experts and scholars in the fields of surgery, medical oncology, radiotherapy, imaging, pathology, and pharmacy for breast cancer diagnosis and treatment. Since its establishment, the committee has been committed to strengthening the management and control of breast cancer medical quality, standardizing breast cancer diagnosis and treatment activities in medical institutions at all levels, and improving the level of breast cancer diagnosis and treatment nationwide. After the establishment of this expert committee and a number of national guidelines and norms (covering the entire course of breast cancer diagnosis and treatment, including the NHC's *Guidelines for Diagnosis and Treatment of Breast Cancer*, *Guidelines for Rational Drug Use for Breast Cancer*, *Chinese Breast Cancer Screening and Early Diagnosis and Early Treatment Standards*, *Guidelines for Standardized Diagnosis and Treatment of Advanced Breast Cancer in China*, and *Guidelines for Follow‐up and Health Management of Breast Cancer in China*), the top‐level framework for breast cancer quality control management is mostly complete.

On this basis, starting from 2019, the expert committee focused on system construction and promoted the implementation of specific measures for quality control management. Regarding organizational system construction, the expert committee focused on three tasks: (1) establishment of a provincial‐level breast cancer single‐disease quality control expert committee; (2) establishment of a breast cancer single‐disease quality control subspecialty group; and (3) the construction of quality control pilot projects for standardized diagnosis and treatment of breast cancer. With the help of the NCC, these three tasks have been carried out from top to bottom and have received nationwide positive responses. To date, more than 20 provinces have established provincial‐level expert committees for the single‐disease quality control of breast cancer. Through the national breast cancer single‐disease quality control system, the special committee has established as well as promoted the construction of seven subspecialty quality control systems for radiological diagnosis, ultrasonic diagnosis, pathological diagnosis, internal medicine treatment, surgical treatment, radiotherapy, and pharmaceutical management.

## 2 QUALITY CONTROL PILOT WORK ON STANDARDIZED DIAGNOSIS AND TREATMENT OF BREAST CANCER

In September 2019, the NCC created the Pilot Project on Quality Control of Standardized Diagnosis and Treatment of Breast Cancer, aimed at strengthening the standardized management of diagnosis and treatment of single‐tumor diseases, establishing and improving the national standardized breast cancer diagnosis and treatment and quality control system, and improving the overall level of diagnosis and treatment of breast cancer nationwide. In a short period of time, nearly 1000 hospitals submitted applications for this pilot program. Upon selecting the pilot centers, the expert committee reviewed applicants from multiple perspectives, including the degree of discipline, academic influence, medical conditions, and relevant monitoring data. In December 2020, in accordance with the principles of fairness, justice, science and rationality, and relevant selection conditions, the NCC and the National Cancer Quality Control Center Breast Cancer Expert Committee selected 200 hospitals as pilot centers. These quality control pilot units cover 30 provinces (autonomous regions and municipalities) across the country excluding Tibet. The special committee is currently evaluating the first batch of breast cancer standardized diagnosis and treatment pilot projects and will award the National Cancer Quality Control Center Breast Cancer Standardized Diagnosis and Treatment Quality Control Standard Center/Demonstration Center to the pilot units that have passed the quality control during the construction period. The application for the second batch of pilot units has also begun.

## 3 INCREASING INFORMATIZATION OF BREAST CANCER QUALITY CONTROL

Quality control cannot begin without accurate data. To implement the requirements of the national quality control informatization work, the NCC adheres to the principle of “one source for multiple purposes; platform sharing,” and has used the NCC's National Antitumor Drug Clinical Application Monitoring Network to establish a Breast Cancer Special Disease Database. This database includes 19 types of data tables, 25 forms, and 1509 fields, including information on patient‐outpatient, hospitalization, drug, medical technology, treatment, and follow‐up. Furthermore, the quality control center requires breast cancer quality control pilot units to report data to the monitoring platform via automatic docking within the monitoring range of the national antitumor drug clinical application monitoring network. Through the joint efforts of the monitoring network and pilot units, all the first batch of 200 pilot units have become monitoring units of the National Antitumor Drug Clinical Application Monitoring Network as of July 31, 2023, and dozens of units have begun reporting data to the monitoring network through automatic docking.

## 4 BREAST CANCER SINGLE‐DISEASE QUALITY CONTROL INDEX

To further promote the quality control of breast cancer diagnosis and treatment, the NCC and the National Cancer Quality Control Center commissioned the Breast Cancer Expert Committee to follow the *Breast Cancer Diagnosis and Treatment Guidelines (2022 Edition)* and other national breast cancer diagnosis and treatment guidelines, as well as evidence‐based medical evidence, expert recommendations, clinical experience, and China's national conditions. In line with the guiding principles of scientificity, normativeness, universality, and operability, the Expert Committee published the *Quality Control Index for Standardized Diagnosis and Treatment of Breast Cancer in China (2022 Edition)* in March 2022 [4]. The index includes 20 quality control indexes and four quality management indexes, including the clinical tumor, node, and metastasis (TNM) staging evaluation rate before the first treatment of breast cancer patients, covering diagnosis, surgery, drug treatment, radiotherapy, and other links, thus providing a basis for the development of quality control work.

To date, China's breast cancer single‐disease quality control, while starting from scratch, has conducted substantial pioneering work and set an example for other tumor types. The Breast Cancer Quality Control Expert Committee has formulated national uniform quality control indexes for breast cancer, carried out the pilot work of standardizing breast cancer diagnosis and treatment, focused on quality control indexes to establish and improve the national breast cancer quality control system based on the pilot units, and promoted the normalization, homogeneity, and standardization of breast cancer diagnosis and treatment nationwide.

Going forward, the Breast Cancer Quality Control Expert Committee will continue to expand the coverage of pilot work, accept the first batch of pilot centers and carry out the second batch of pilot center declarations, promote the expansion of high‐quality medical resources, and continue to improve the breast cancer prevention and control capabilities of provincial‐, municipal‐, and county‐level medical institutions nationwide through quality control work. This will ultimately improve the survival rate and quality of life of breast cancer patients in China.

## AUTHOR CONTRIBUTIONS

**Bo Lan**: Writing—original draft (lead). **Qiao Li**: Writing—review and editing (supporting). **Fei Ma**: Conceptualization (equal); Project administration (equal); Supervision (equal). **Binghe Xu**: Conceptualization (equal); Project administration (equal); Supervision (equal).

## CONFLICT OF INTEREST STATEMENT

Professor Binghe Xu and Fei Ma are members of the *Cancer Innovation* Editorial Board. To minimize bias, they were excluded from all editorial decision‐making related to the acceptance of this article for publication. The remaining authors declare no conflict of interest.

## ETHICS STATEMENT

Not applicable.

## INFORMED CONSENT

Not applicable.
